# Design Thinking: An Interprofessional Method for Finding Comfort in the Discomfort Surrounding End-of-Life Conversations Between Providers and Patients

**DOI:** 10.1007/s40670-025-02374-z

**Published:** 2025-04-01

**Authors:** Tracey Thurnes, Anna Foster, Dianne Person

**Affiliations:** Anatomical Gift Program, Department of Physician Assistant Studies, School of Health Sciences, Elon University, Elon, NC 27244 USA

**Keywords:** End-of-life care, Advanced care planning, Goals of care, Design thinking

## Abstract

This paper discusses the development of a novel curriculum utilizing design thinking practices to train physician assistant (PA) students in conducting end-of-life (EOL) conversations. The curriculum aims to equip future healthcare providers with the necessary communication skills for advanced care planning (ACP) and goals of care (GOC) discussions, which are crucial for respecting patient autonomy and improving care quality. This training program, which includes utilization of the design thinking framework, role-playing, and community volunteer involvement, addresses the current gap in EOL conversation training. The anticipated outcomes of integrating structured strategies for difficult conversations in medical education include enhanced patient autonomy and bolstered provider confidence, contributing to improved clinical communication and decision-making efficacy.

## Background

A patient’s autonomy during the end stages of life is a crucial component of healthcare. Advanced care planning (ACP) involves essential conversations between providers and patients about future care plans after a serious illness diagnosis. These discussions are ongoing dialogues, not one-time events. Providers often struggle with timing and initiating these conversations, leading to crucial end-of-life discussions occurring when patients can no longer make decisions [1–3].

Studies show that the lack of training for healthcare professionals in goals of care (GOC) and ACP conversations increases the risk of adverse patient outcomes [4–6]. Training is essential to enhance patient autonomy, improve care quality, and provide better outcomes for patients and providers. With the growing number of physician assistant (PA) programs and the increased need for advanced practice providers (APPs), a novel curriculum was developed for PA students. This program uses design thinking practices and community volunteers to equip future healthcare providers with the necessary tools and communication skills for end-of-life conversations.

## Activity

The training program integrates a design thinking framework, role-playing, and community volunteer engagement to prepare students for ACP and GOC discussions. Design thinking, an interactive and collaborative process involving empathizing, defining, ideating, prototyping, and testing, serves as the foundation for training second-year PA students. Originally developed in fields like engineering and product design, the design thinking framework has been increasingly applied in healthcare to address complex challenges, improve patient experiences, and enhance communication between providers and patients [7, 8].

Students were provided structured guidelines for conducting GOC conversations, including strategies for setting up discussions, assessing patient understanding and preferences, sharing prognosis, and exploring key topics such as goals, fears, and care priorities [9, 10]. The training aimed to create a supportive space for exploring difficult conversations; build student confidence in end-of-life discussions; foster humility, empathy, compassion, and respect; and equip both students and aging adults with essential communication skills and resources.

The program has evolved through multiple iterations to adapt to changing circumstances while enhancing outcomes. The initial pilot was a four-hour in-person session where second-year PA students partnered with community members for role-playing, reflective exercises, and group discussions using tools like empathy maps and structured debriefing models. During the pandemic, the program transitioned to a virtual format, preserving its core objectives through online breakout rooms, facilitated discussions, and tailored debriefs that emphasized the humanization of difficult conversations.

After the pandemic, the training program resumed in person while adhering to health and safety guidelines. Building on insights from previous models and interdisciplinary education research, the session expanded into a fully interprofessional event [11, 12]. It included second-year PA students, accelerated nursing students, faculty from both disciplines, and hospice and palliative care educators. This iteration aimed to improve learning outcomes by encouraging interdisciplinary discussions on the roles of both patients and healthcare providers in navigating difficult conversations.

*Session description:*

The three-hour agenda began with an introduction period, followed by a keynote address from a local hospice educator. Participants then engaged in an empathy mapping exercise, a tool used in the empathy phase of design thinking, to reflect on their initial thoughts and experiences with ACP and GOC discussions. A facilitated discussion with the larger group helped identify overarching themes that emerged from the silent reflections.

Students and community members were then divided into small interdisciplinary groups consisting of PA students, nursing students, and two community members. These groups engaged in a 45-min discussion around ACP and GOC planning, with conversation prompts provided as needed. Role-playing exercises were incorporated to simulate real-world scenarios, followed by structured feedback from community members and peers, allowing participants to refine their approach to these conversations. The session concluded with a large-group reflection followed by small-group debriefs led by faculty educators.

Each version maintained a focus on creating a supportive environment, leveraging design thinking, and cultivating empathy, demonstrating its adaptability and effectiveness in advancing ACP and GOC conversations.

## Results

Participants reported increased confidence and comfort in conducting end-of-life (EOL) conversations, emphasizing the workshop’s effectiveness in mitigating barriers such as fear and hesitation in initiating these discussions. Many highlighted the program’s potential to improve clinical communication surrounding EOL care by providing structured guidance and opportunities for practice.

A total of 48 participants, including PA students, nursing students, and community volunteers, completed an anonymous post-program survey assessing the effectiveness of the in-person training program. Respondents rated three statements using a Likert scale: (1) “The workshop better equipped me to have difficult conversations,” (2) “The workshop instructions were easy to follow,” and (3) “I was highly engaged during the workshop.”

In response to the first statement, 50% of participants strongly agreed, 39.58% agreed, and only 2.08% strongly disagreed. For the second statement, 97.91% of respondents either strongly agreed or agreed that the instructions were easy to follow. Regarding engagement, 93.75% of participants reported feeling highly engaged.

In addition to survey responses, 24 community volunteers participated in a separate debrief session, where discussions indicated a greater sense of preparedness for EOL conversations. Many participants described their experience with terms such as “helpful,” “informative,” “humbling,” and “gratitude,” underscoring the workshop’s impact on fostering confidence and empathy in difficult discussions. One participant noted, “The sessions were inspiring as we shared our personal sensitive end of life thoughts with the PA students. As senior citizens we provided personal insights and values that were developed over many years. In return, the interactions were so open, and we felt a genuine sense of camaraderie with the students.”

The integration of design thinking principles and the inclusion of community volunteers provided a structured yet adaptable framework that enhanced engagement and reinforced the development of key communication skills. The findings highlight the value of incorporating structured training strategies into medical education to enhance provider confidence, promote patient-centered care, and improve interdisciplinary communication in challenging conversations.

## Discussion

Incorporating standardized EOL, GOC, and ACP training programs in healthcare curricula is crucial for improving patient outcomes. By equipping PA students with the necessary communication skills, this novel curriculum addresses a vital need in healthcare education. The training program emphasizes respect for patient autonomy during the end stages of life and focuses on improving practitioner knowledge and confidence. Feedback also indicated a preference for earlier integration of the program or the inclusion of multiple workshops throughout the clinical year to provide more opportunities to practice and apply the skills gained. These insights will inform future adjustments to optimize the program’s timing and participation.

## Conclusion

PAs are uniquely positioned to lead ACP and GOC discussions with their patients. This innovative curriculum equips practitioners with the knowledge, confidence, and respect for patient autonomy necessary to navigate these challenging conversations effectively.

By continuously refining and enhancing this training program, it addresses a critical gap in healthcare education, serving as a model for other programs seeking to improve end-of-life communication training. Such curricular advancements have the potential to elevate clinical communication, foster better decision-making, and ultimately support more meaningful and impactful provider-patient relationships.
